# Regulatory Mechanism and Experimental Verification of Patchouli Alcohol on Gastric Cancer Cell Based on Network Pharmacology

**DOI:** 10.3389/fonc.2021.711984

**Published:** 2021-09-01

**Authors:** Yanru Song, Liang Chang, Xiaoyuan Wang, Bibo Tan, Jianbo Li, Jie Zhang, Fengbin Zhang, Lianmei Zhao, Guangjie Liu, Bingjie Huo

**Affiliations:** ^1^Department of Traditional Chinese Medicine, The Fourth Hospital of Hebei Medical University, Shijiazhuang, China; ^2^Department of Basic Theory of Traditional Chinese Medicine of Basic Medical Science College, HeBei University of Chinese Medicine, Shijiazhuang, China; ^3^Department of General Surgery, The Fourth Hospital of Hebei Medical University, Shijiazhuang, China; ^4^Department of Gastroenterology Pharmacology, The Fourth Hospital of Hebei Medical University, Shijiazhuang, China; ^5^Research Centre, The Fourth Hospital of Hebei Medical University, Shijiazhuang, China; ^6^Department of Thoracic Surgery, The Fourth Hospital of Hebei Medical University, Shijiazhuang, China

**Keywords:** gastric cancer, network pharmacology, proliferation, apoptosis, Patchouli alcohol

## Abstract

**Background:**

*Pogostemon cablin* is a traditional Chinese medicine (TCM) that is frequently used to treat various gastrointestinal diseases. Patchouli alcohol (PA), a compound extracted from the *Pogostemon cablin*, has been shown to have anti-tumor efficacy in human colorectal cancer. However, the mechanism of PA’s anticancer effect on gastric cancer (GC) remains unknown.

**Methods:**

We used the public database to obtain the potential targets of PA and genes related to GC. Bioinformatic analyses, such as the Kyoto Encyclopedia of Genes and Genomes (KEGG), Gene Ontology (GO), and protein-protein interactions (PPI), were used for analyzing the potential signal pathways and targets. Cell experiments were also conducted to further explain the impact and molecular mechanism of PA on GC, as well as to confirm the findings of network pharmacology.

**Results:**

Using network pharmacological analysis, 161 possible targets were identified for the treatment of GC. Network analysis and functional enrichment analysis show that PA produced a marked effect in the treatment of GC through multi-targets and multi-pathways, especially the MAPK and PI3K/AKT signal pathways. In addition, PA showed the inhibition of GC cell proliferation, migration and invasion in cell experiments. According to our findings, PA could also cause G0/G1 phase arrest and apoptosis in GC cells.

**Conclusion:**

Using network pharmacology, we aim to uncover the possible molecular mechanism of PA on GC treatment in this research. Cell experiments were also conducted to confirm the therapeutic effect of PA on GC.

## 1 Introduction

Gastric cancer (GC) is the world’s fifth most common cancer and the third leading cause of deaths related to cancer (1). Patients with early GC are generally asymptomatic and most of them are diagnosed in the late stage (2). Although surgery, chemotherapy, radiotherapy, and targeted drugs have made great progress in the treatment of GC, the rate of mortality in GC patients is still high due to tumor recurrence and metastasis. The 5-year survival rate of GC has been reported to be less than 20% (3). Therefore, it is necessary to explore new strategies for GC treatment.

Traditional Chinese medicines (TCM) have gotten a lot of recognition as a vital part of complementary and alternative medicine, and they played an important role in cancer prevention and treatment (4). Pogostemonis Herba, a dried aerial part from *Pogostemon cablin* (Blanco) *Benth* (Labiatae), has traditionally been used to treat gastrointestinal diseases. Patchouli alcohol (PA) is a tricyclic sesquiterpene isolated from the *Pogostemon cablin* plant that has a variety of therapeutic activities such as anti-inflammation (5), anti-gastro ulcerogenic (6), antimicrobial (7), protection against the intestinal epithelial cells fluidity (8), and so on. It is reported that PA has protective effects on oxidative stress, apoptosis, and inflammation of human gastric epithelial cells induced by Helicobacter pylori (9). Since inflammation is linked to the development of GC, it’s reasonable to believe that PA could have anticancer properties in this disease.

PA’s anticancer activity and potential mechanism have recently been demonstrated in some studies. PA prevented the growth and caused concentration-dependently apoptosis in human colorectal cancer cells (SW480 and HCT116) (9). In addition, PA has been shown to inhibit the HeLa cells proliferation *in vitro* (10). PA can also inhibit the A549 cell’s growth both *in vivo* and *in vitro*, as well as induce apoptosis and stop cell cycle progression (11). The anticancer effect of PA on human GC, on the other hand, has not been documented.

Therefore, in this study, the network pharmacology method was used to analyze PA, construct a PPI network of PA against GC, and mine the dominating biological functions and signaling pathways. Finally, the effects and enriched key signaling pathway were verified by *in vitro* cell experiments, which provide new reference information for the development and application of PA. Although all the results of this study have proved the anti-cancer effect of PA on GC, the limitation of our study is that the molecular mechanism of this study was simple, only a series of assays *in vitro* were carried. In future, more *in vivo* experiments are needed to support our findings, and future studies should focus on providing experimental evidence and expanding the role of PA in GC.

## 2 Materials and Methods

### 2.1 Network Pharmacology

#### 2.1.1 Access to Disease Genes and Drugs Targets

**Figure 1:**
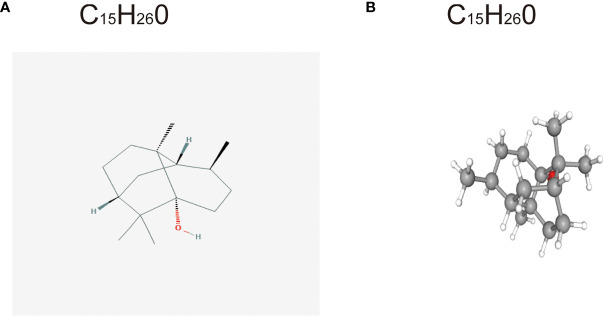
**(A)** 2D structure of PA. **(B)** 3D structure of PA.

**Figure 2:**
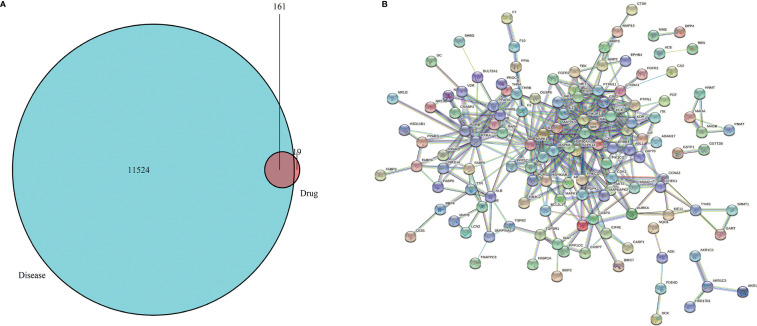
**(A)** Venn diagram. The green represents the GC targets, the red represents PA targets, and the shaded part represents the intersection targets of PA and GC. **(B)** Target proteins interaction network. The nodes in the network represent proteins, the lines show the protein’s functional associations, and the thickness of lines corresponds to the observed association’s confidence level.

The 2D and 3D structures (**Figures 1A, B**) of PA were obtained from PubChem (https://pubchem.ncbi.nlm.nih.gov/), and the files of 3D structures were uploaded to PharmMapper (http://www.lilab-ecust.cn/pharmmapper/), according to the parameters set in the literature: generate conformation: yes; maximum generated conformation: 300; selected target set: only human protein target (v2010, 2241); reserved number of matching targets: 300. Then, the GC-related targets were obtained from GeneCards (https://www.genecards.org/) and the Online Mendelian Inheritance in Man (OMIM) database (http://omim.org/). Finally, we used the “VennDiagram” software package of R software to identify common targets from PA potential targets and GC targets and draw Venn diagram (**Figure 2A**).

#### 2.1.2 Protein-Protein Interaction Network

To obtain the Drug-Disease Interaction Network (PPI), the common targets were imported into the STRING database (https://string-db.org). The parameters were set as following: species: “Homo sapiens”, the highest confidence: “scoring value > 0.9”, “hide network disconnected nodes”, and output files in TSV format. Then, the TSV file was imported into the Cytoscape 3.7.0 and the PPI network was arranged according to the degree value. Different colors and sizes of the circle represent degrees. The closer to the center, the darker the color, the higher the degree.

#### 2.1.3 Gene Function and KEGG Enrichment Analysis

The Bioconductor software package “org.hs.eg.db” was installed in R software and ran it to convert the drug-disease common target”. The common target was then converted into the entrez ID. Then, the “clusterProfiler” package was installed in R software, and enrichment analyses of GO (12) and KEGG (13) about the key target genes with P<0.05 and Q<0.05 according to the converted “entrezID”. The results were output in the form of a bar chart. In addition, the annotation of the target was obtained by KEGG enrichment analysis, and it was visualized by Cytoscape 3.7.0.

### 2.2 Experimental Analysis

#### 2.2.1 Cell Culture

The Cell Bank of the Chinese Academy of Sciences, Shanghai Institute of Cell Biology (Shanghai, China), provided the GC cell lines NCI-N87 and HGC-27. The cells were grown in RPMI-1640 medium with foetal bovine serum (10%, FBS) (HyClone, China) and a mixture of 1% antibiotics (streptomycin 100 μg/mL, penicillin 100U/mL) in an incubator containing 5% CO_2_ at 37°C.

#### 2.2.2 MTT Assay

NCI-N87 and HGC-27 cells were digested with trypsin to make single-cell suspension followed by their seeding in a 96-well plate at 5 × 10^4^ cells/well. GC cells were incubated with different concentrations of PA (0 μ M, 0.5 μ M, 1 μ M, 2 μ M) in a constant temperature incubator at 37°C for 24 h, 48 h and 72 h, respectively. After the end of the culture, 20 μ L MTT (5mg/mL) was added to each well for 2 hours. The blank control hole was adjusted to zero, and the OD value was determined at 490 nm by enzyme labeling instrument.

#### 2.2.3 Investigation of Apoptosis and Cell Cycle Through Flow Cytometry

HGC-27 and NCI-N87 GC cells were inoculated into a 6-well plate and were subjected to treatment with PA (1 μ M). PA (0 μ M) was used as the control group. The cells of each group were collected after 48 hours of corresponding treatment, washed, and centrifuged. According to the production instructions of AnnexinV/PI apoptosis kit, 500 μL buffer solution was added to resuscitate the cells, and then 5 μL of each PI and Annexin V-FITC were added and left for 15 min at room temperature. Flow cytometry was used to detect apoptosis.

The cells of each group after corresponding treatment for 48 hours were collected again, washed twice with pre-cooled PBS washing solution, and fixed with 70% cold ethanol for overnight use. This was followed by the addition of RNase A of 50 mg/mL and PI of 50 mg/mL and left for incubation at 37°C for 30 min. The cell cycle was subsequently investigated by Flow cytometry.

#### 2.2.4 Wound Healing Assay

A wound healing assay was used for performing the cell migration assay. HGC-27 and NCI-N87 GC cells were inoculated into 6-well plates. After the cells were completely fused, the monolayer was scratched with the pipette tip of 10 μ L straw and washed with PBS to remove cell fragments. Then treated with different concentrations (0 μ M, 1 μ M) of PA for 48h. The scratches were imaged with a 10-fold objective lens at 0 and 48 hours after the scratch. The cell migration rate and the distance between the healed wound and the original wound were calculated.

#### 2.2.5 Transwell Assay

NCI-N87 and HGC-27 cells were inoculated in 24-well plates and incubated overnight in the incubator. Then, different concentrations of PA (0 μ M, 1 μ M) were added according to the experimental groups. Trypsin was used for the digestion of cells in each group and the cell suspensions were prepared with a concentration of 10^3^ cells/ml. The upper chamber of a Transwell coated with Matrigel was filled with cell suspension (100 μL), while the lower chamber was filled with DMEM culture medium containing 10% foetal bovine serum. Cells were washed three times in PBS after 48 hours of culture in the incubator, and the cells in the chamber were carefully wiped with cotton swabs. This was followed by their fixation and staining with paraformaldehyde (4%) and crystal violet (0.1%), respectively. There were 3 multiple holes in each group, and 5 high-power lens visual fields were randomly taken under an inverted microscope for each hole. The number of transmembrane cells was counted. The experiment was repeated 3 times.

#### 2.2.6 Western Blotting

The cells were lysed with RIPA lysate and centrifuged after being treated with different PA concentrations (0 μM, 1 μM) for 48 hours. The concentration of protein in the supernatant was measured using a BCA kit. The separation of the total proteins from 40 μg samples was carried out with SDS-PAGE electrophoresis. The separated proteins were then electroporated onto PVDF membranes. Then, the PVDF membrane was sealed with TBS solution containing 5%BSA for 1 hour, and incubated with the appropriate primary antibody solution including PCNA, CyclinD1, Bcl-2, Bax, caspase3, MMP2, MMP9, PI3K, p-PI3K, AKT, p-AKT, ERK, p-ERK, P38, p-P38, JNK, p-JNK, β-actin (diluted at 1: 1,000) overnight at 4°C. The membrane was washed three times with TBST the next day, each time for five minutes, and then the second antibody was added to incubate for 2 hours at room temperature. The membrane was again subjected to washing with TBST, colored and exposed by ECL, developed, fixed, photographed, and analyzed.

### 2.3 Statistical Analysis

The data between groups were analyzed using one-way ANOVA (GraphPadPrism 7 software). For the data between the two groups, we performed the Student’s *t*-test. These results were presented as the mean ± standard error of the mean (SEM) for the treated experimental and control groups. *P-values* < 0.05 were regarded as statistically significant.

## 3 Results

### 3.1 Targets Fishing

We obtained the 2D and 3D structures of PA from PubChem database (**Figures 1A, B**). Then we predicted potential binding genes of PA using the PharmMapper platform. Finally, 180 genes were predicted to be PA-binding genes. Afterwards, from OMIM and GeneCards database, there were 11,524 GC-related target genes. Finally, we executed the venny.R command and extracted 161 common targets from the two documents, the green represents the GC targets, the red represents PA targets, and the overlap region represents the intersection targets of PA and GC (**Figure 2A**).

### 3.2 PPI Network Analysis

**Figure 3:**
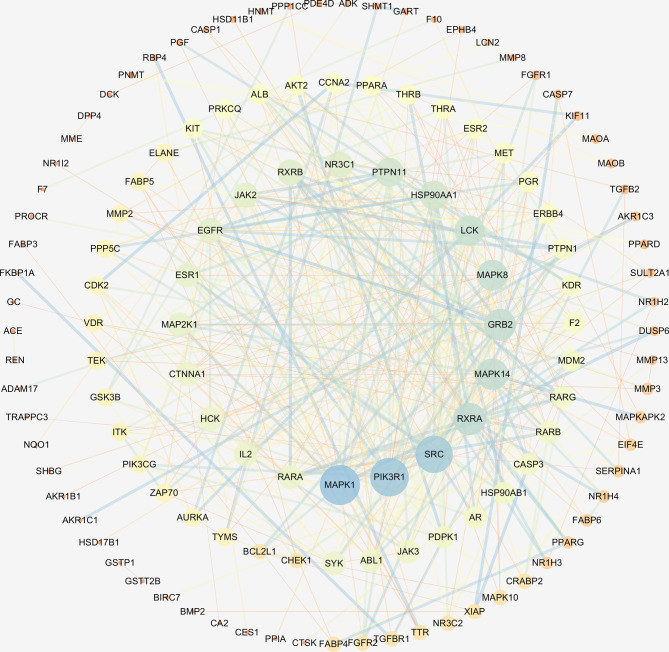
The hub genes of PPI network. Higher degrees indicated larger node sizes, and blue indicates higher degree, and orange represents the lowest degree.

The interaction network had 161 nodes and 168 edges (**Figure 2B**). The TSV data were imported into Cytoscape, the results show that there are 129 nodes and 425 edges, and the PPI value < 1.0e-16. In this network, proteins are represented as nodes with different colors, and the lines connecting the nodes represent functional associations between proteins. Line thickness indicates the strength of confidence in the reported association. For a node, the number of connections of the node is the degree of the node. The higher the degree, the more the gene is in a position in the regulatory network, indicating that it is a hub gene. The top ten hub genes were MAPK, PIK3R1, SRC, RXRA, MAPK14, GRB2, MAPK8, LCK, HSP90AA1, PTPN11 (**Figure 3**).

### 3.3 GO and KEGG Enrichment Analysis

**Figure 4:**
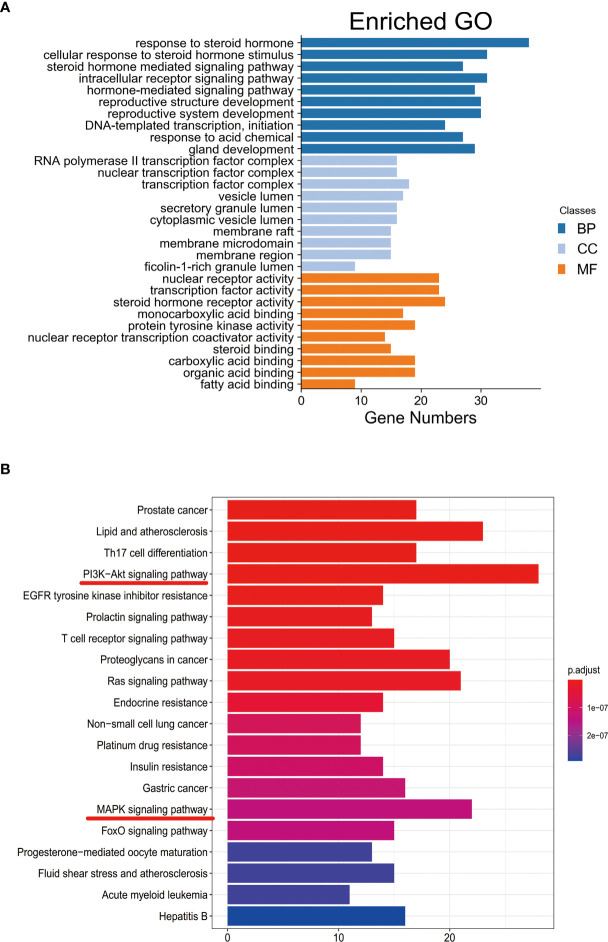
**(A)** GO enrichment analysis. The longer the bar chart is, the higher the enrichment degree of each biological process, biological processes are represented by blue, cellular components are represented by light blue, and molecular function is represented by orange. **(B)** KEGG enrichment analysis. The larger the bar plot diagram, the higher the enrichment degree of each KEGG pathway and the color of the bar plot map represents the gene enrichment of each KEGG pathway.

**Figure 5:**
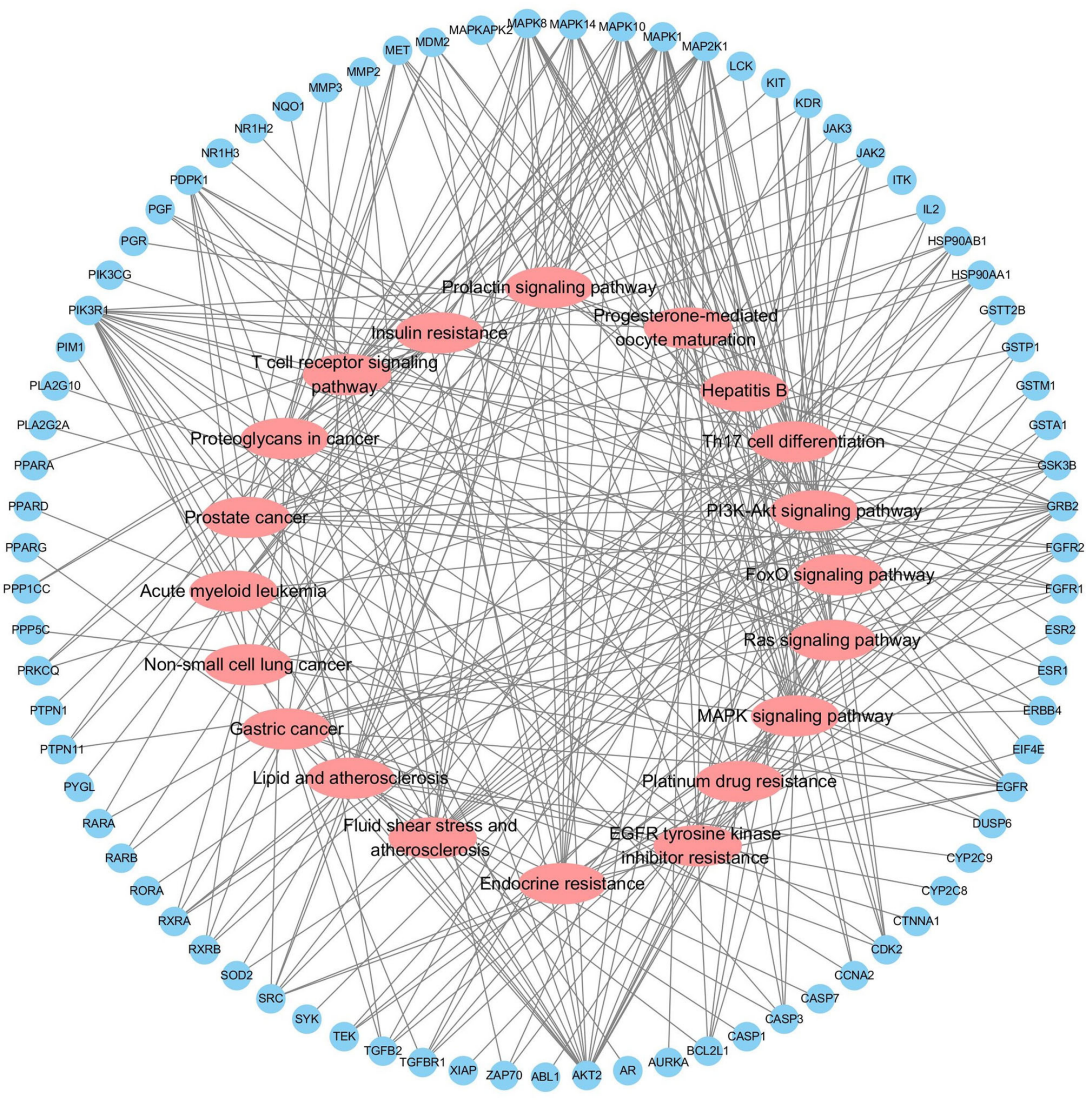
Target-Pathway Network. The target nodes are represented by the blue ellipse nodes. The corresponding pathways are represented by the red diamond nodes.

Functional enrichment analysis for the target genes was used for further investigating the PA mechanism on GC. The GO functional enrichment results (**Figure 4A**) showed that PA was related to nuclear transcription factor complex, RNA polymerase II transcription factor complex, vesicle lumen, and transcription factor complex. Moreover, PA may play a regulatory role in transcription factor activity, steroid hormone receptor activity, direct ligand regulated sequence-specific DNA binding, nuclear receptor activity, and monocarboxylic acid-binding. PA may also participate in cells’ responses to steroid hormone stimulus, a steroid hormone, pathway of intracellular receptor signaling, and steroid hormone-mediated signaling pathway. The findings of the KEGG pathway analysis (**Figure 4B**) revealed that PA might regulate GC progression through multiple pathways related to cancer, including Ras signaling pathway, PI3K-Akt signaling pathway, Th17 cell differentiation, and Proteoglycans in cancer. The PI3K-Akt signaling pathway was the most common among them. To elucidate the mechanism responsible for PA effects on GS in detail, the construction of a target pathway network was carried out on the basis of the top 20 significant signaling pathways and the genes related to these pathways (**Figure 5**). The constructed network was demonstrated to consist of 99 nodes (79 genes and 20 pathways). Among these pathways, MAPK signaling and PI3K/AKT signaling pathways were the pathways with the highest enrichment degree.

### 3.4 Experimental Validation

#### 3.4.1 PA Inhibited the GC Cells Proliferation

**Figure 6:**
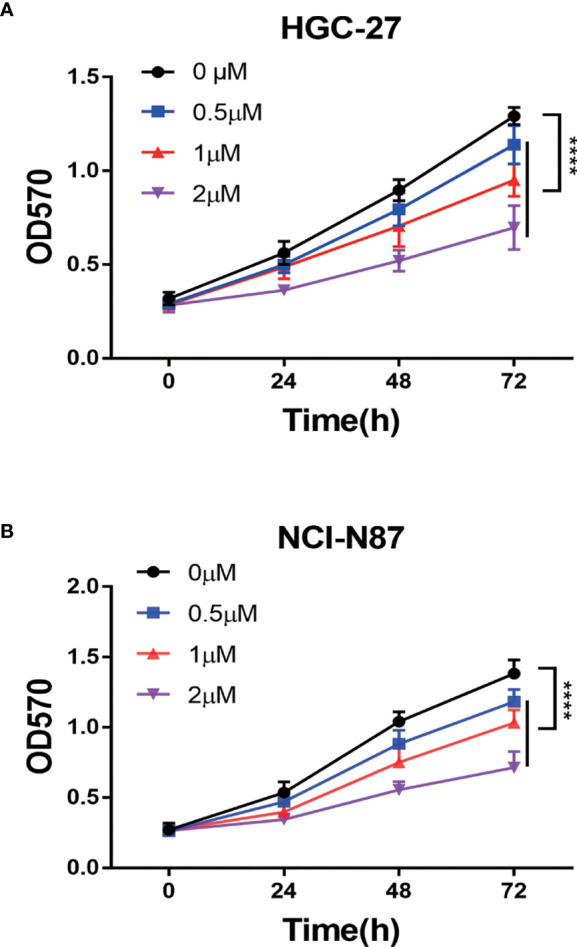
The PA effect on proliferation of cells. **(A)** HGC-27 and **(B)** NCI-N87 cells were treated with various concentrations of PA in a 0-2 µM range for 24 h, 48 h, and 72 h. MTT assay was used for measuring cell viability. (****P < 0.0001).

To confirm PA’s anti-tumor effect on GC, we conducted cell experiments. MTT assay was used for detecting the effect of PA on GC cell proliferation. HGC-27 and NCI-N87 cells were subjected to treatment with different PA concentrations for 24 h, 48 h, and 72 h, respectively (p<0.05). The cell inhibition rate increased in a time- and concentration-dependent manner, according to our findings (**Figure 6**). Furthermore, the PA treatment group’s cell inhibition rate was significantly higher than the control group (p<0.05). These findings indicate that PA was prevent GC cells from proliferating.

#### 3.4.2 PA Induced Apoptosis in GC Cells

**Figure 7:**
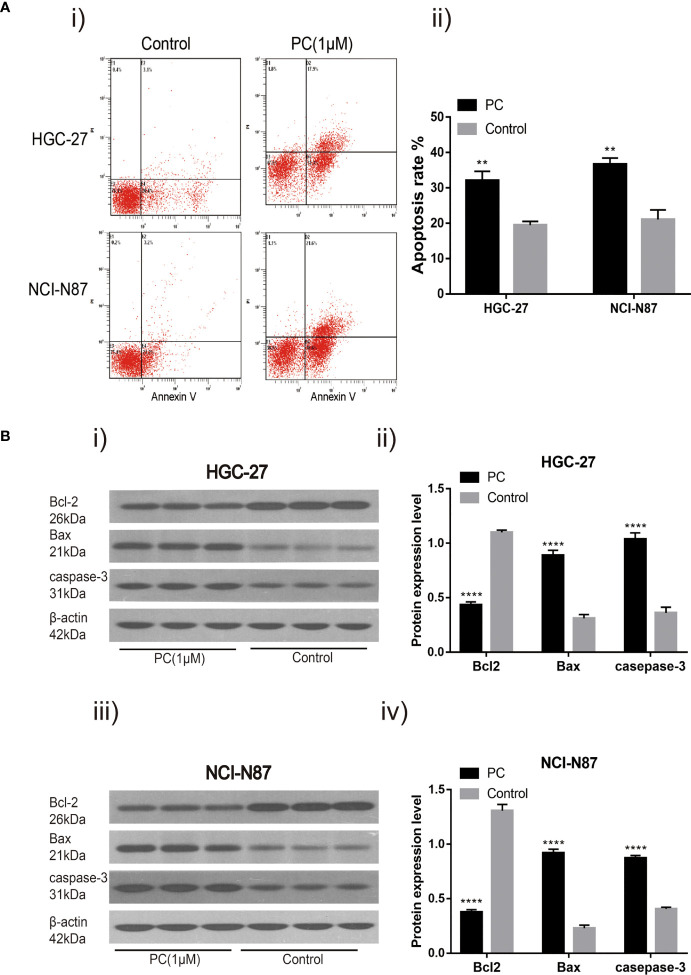
PA induces cell apoptosis. Flow cytometry analysis **(A-i)** of NCI-N87 and HGC-27 cells. After PA treatment, there was a significant increase in the overall rate of apoptosis **(A-ii)**. HGC-27 **(B-i, B-ii)** and NCI-N87 **(B-iii, B-iv)** after being treated with PA for 24 h, the cells were blotted with Caspase-3, Bax, and Bcl2 antibodies. ****P < 0.0001, **P < 0.01. The results were presented as the mean ± SEM of three separate experiments.

We hypothesized that PA caused the inhibition of GC cell proliferation by promoting apoptosis or regulating cell cycle progression. These findings indicate that 1 μM PA effectively prevents GC cells from proliferating. Therefore, a dose of 1 μM was used in subsequent experiments. Flow cytometry was used for detecting the effect of PA on apoptosis in NCI-N87 and HGC-27 cells. The proportion of these cells that went through apoptosis increased significantly after PA treatment as shown by these findings (**Figure 7A**) (p<0.05). Then, to elucidate the molecular mechanism of GC cell apoptosis induced by PA, we measured the expression of apoptosis-related proteins in GC cells treated with PA for 48 h. The Bcl-2 levels in the PA-treated group were lower than those in the control group (p<0.05), according to the findings. The expression of Bax and Caspase-3 was significantly higher in the PA-treated group than in the control group (**Figure 7B**) (p<0.05). We can infer that PA can effectively cause the induction of GC cell apoptosis.

#### 3.4.3 PA Induces Cell Cycle Arrest at G0/G1

**Figure 8:**
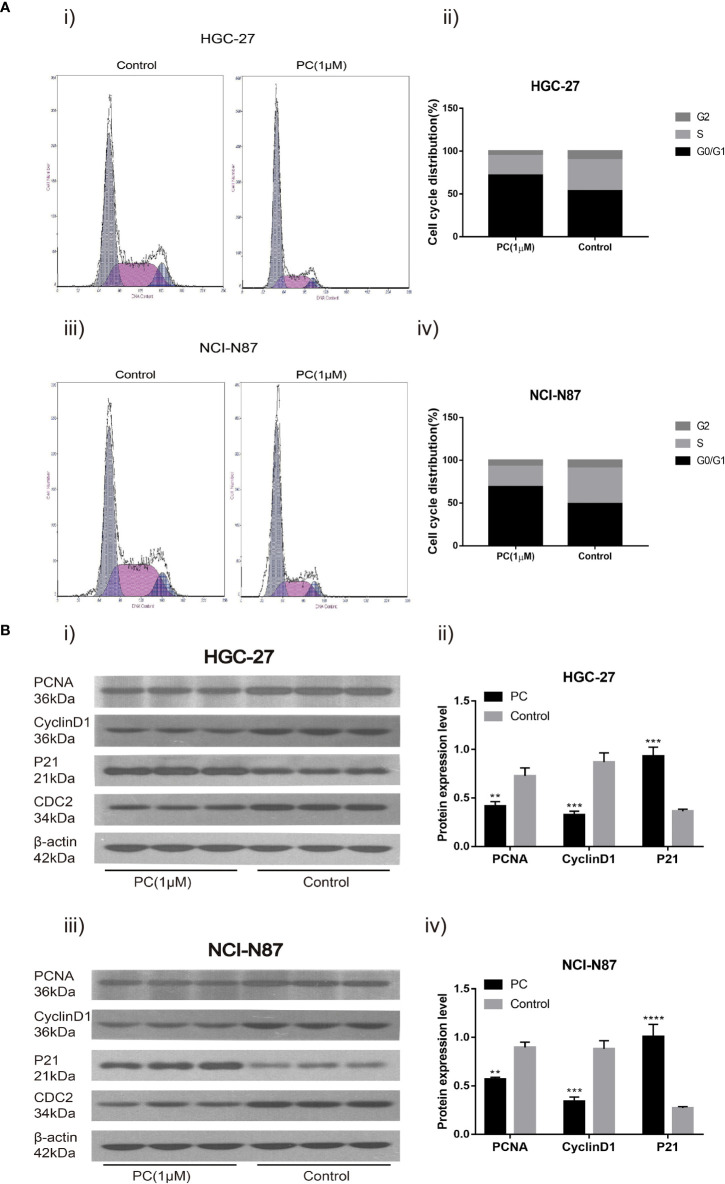
Cell cycle analysis revealed that PA arrested cell growth at the G0/G1 phase. HGC-27**(A-i)** and NCI-N87 **(A-iii)** cells were incubated with a specific dose of PA for 48 h, stained with Propidium iodide, and DNA content of HGC-27 **(A-ii)** and NCI-N87 **(A-iv)** cells was detected via flow cytometry. HGC-27 **(B-i, B-ii)** and NCI-N87 **(B-iii, B-iv)** PA-treated cells were blotted with PCNA, p21, and CyclinD1 antibodies after a 24-hour treatment period. (****P < 0.0001, ***P < 0.001, **P < 0.01).

Flow cytometry was used to detect the influence of PA on the cell cycle distribution of NCI-N87 and HGC-27. The proportion of G0/G1 phase cells in NCI-N87 and HGC-27 cells increased while the proportion of S phase cells decreased in the PA treated group (**Figure 8A**) (p<0.05). In addition, after 48 h of PA treatment, we used Western blot to detect changes in related protein expression. The results showed that in the PA-treated population, the levels of PCNA and CyclinD1 expression were significantly reduced, while the expression of p21 was significantly increased (**Figure 8B**) (p<0.05). We may deduce that PA causes cell cycle arrest in the G0/G1 phase, which has an effect on GC cell proliferation.

#### 3.4.4 PA Inhibits GC Cells Invasion and Migration

**Figure 9:**
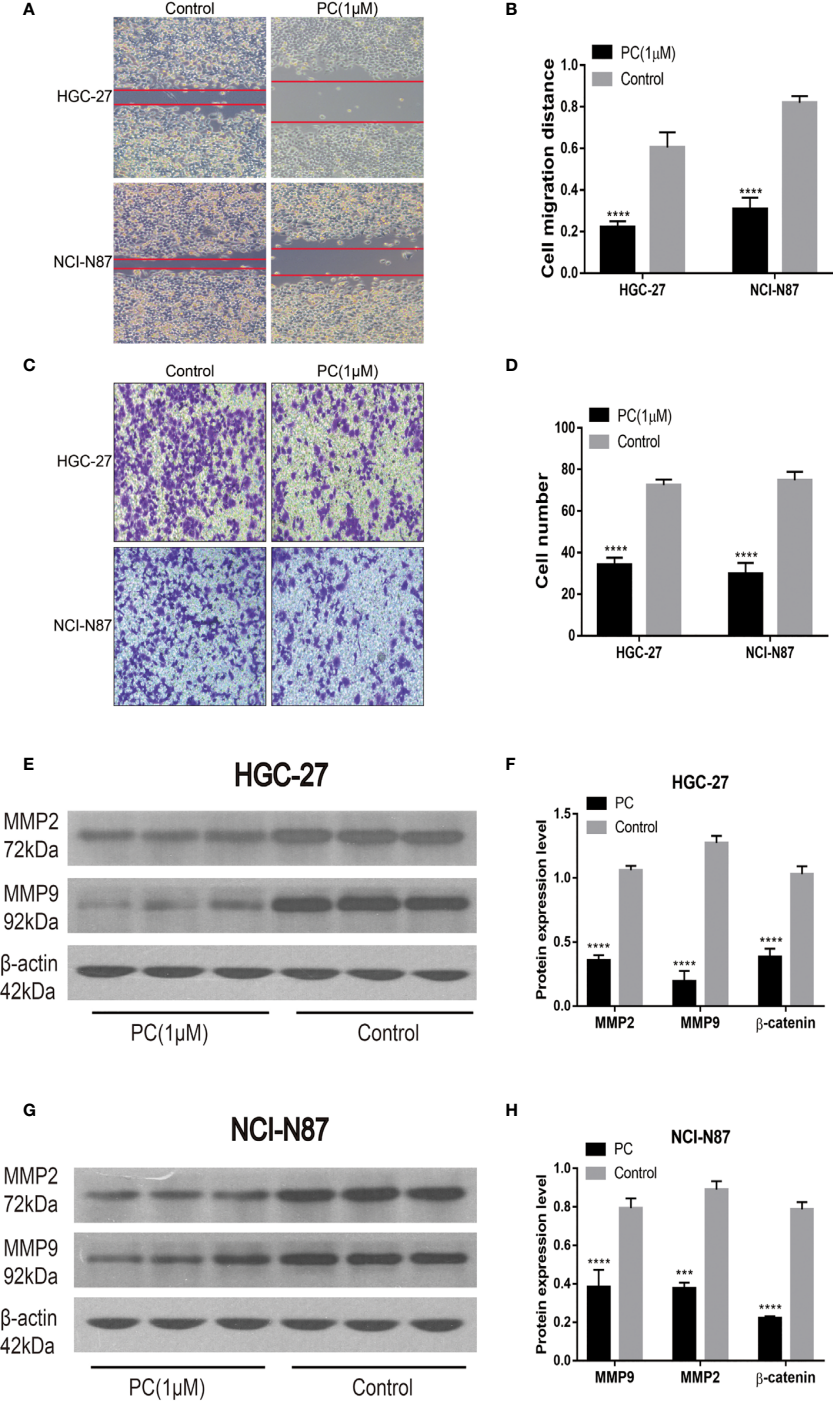
Migration and invasion of HGC-27 and NCI-N87 cells were inhibited by PA. **(A, B)** To assess the PA effects on cell invasion, a transwell assay was used. **(C, D)** Effects of PA on the migration of NCI-N87 and HGC-27 cells *via* wound healing assay. Western blot analysis of MMP2 and MMP9 in HGC-27 **(E, F)** and NCI-N87 **(G, H)** cells. (****P < 0.0001, ***P < 0.001).

Transwell and wound healing experiments were used to investigate PA’s ability to migrate and invade GC cells. Unlike the control group, cell migration was significantly reduced in the PA-treated group, as shown in **Figures 9A** **, B**. Transwell invasion analysis revealed that the PA treatment group’s invasive ability was lower than the control group (**Figures 9C, D**) (p<0.05). In addition, we also used Western blot for investigating the expression of related proteins after 48h of treatment with PA. Our findings revealed that the levels of MMP2 and MMP9 in the PA group were found to be significantly lower (**Figures 9E** **–H**) (p<0.05). We infer that PA can inhibit the GC cell’s invasion and migration.

#### 3.4.5 Pharmacological Authentication of Pathway Enrichment

**Figure 10:**
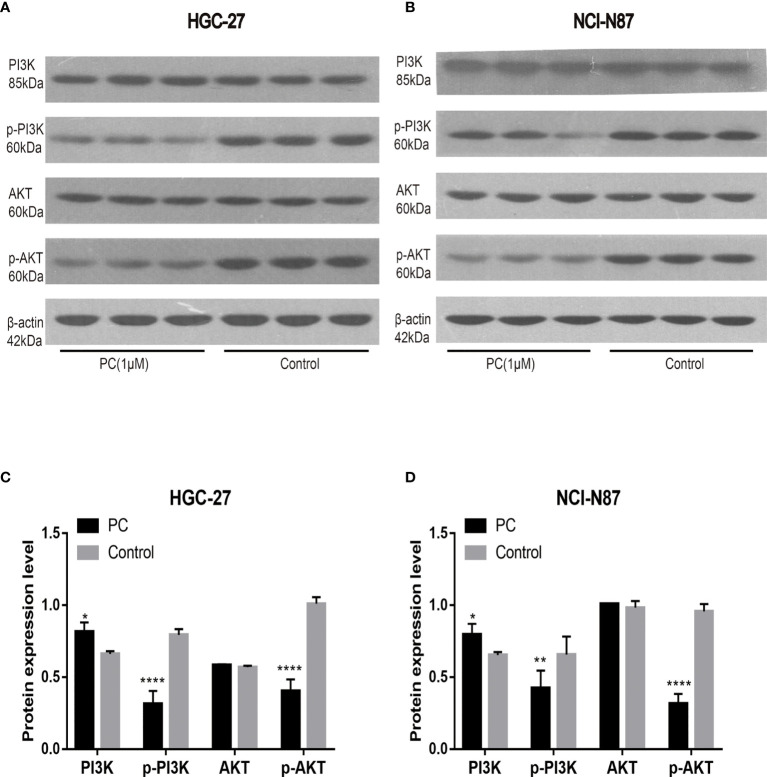
Effects of PA on the expression of proteins related to PI3K/AKT pathway in HGC-27 **(A, C)** and NCI-N87 **(B, D)** cells. (****P < 0.0001, **P < 0.01, *P < 0.05).

**Figure 11:**
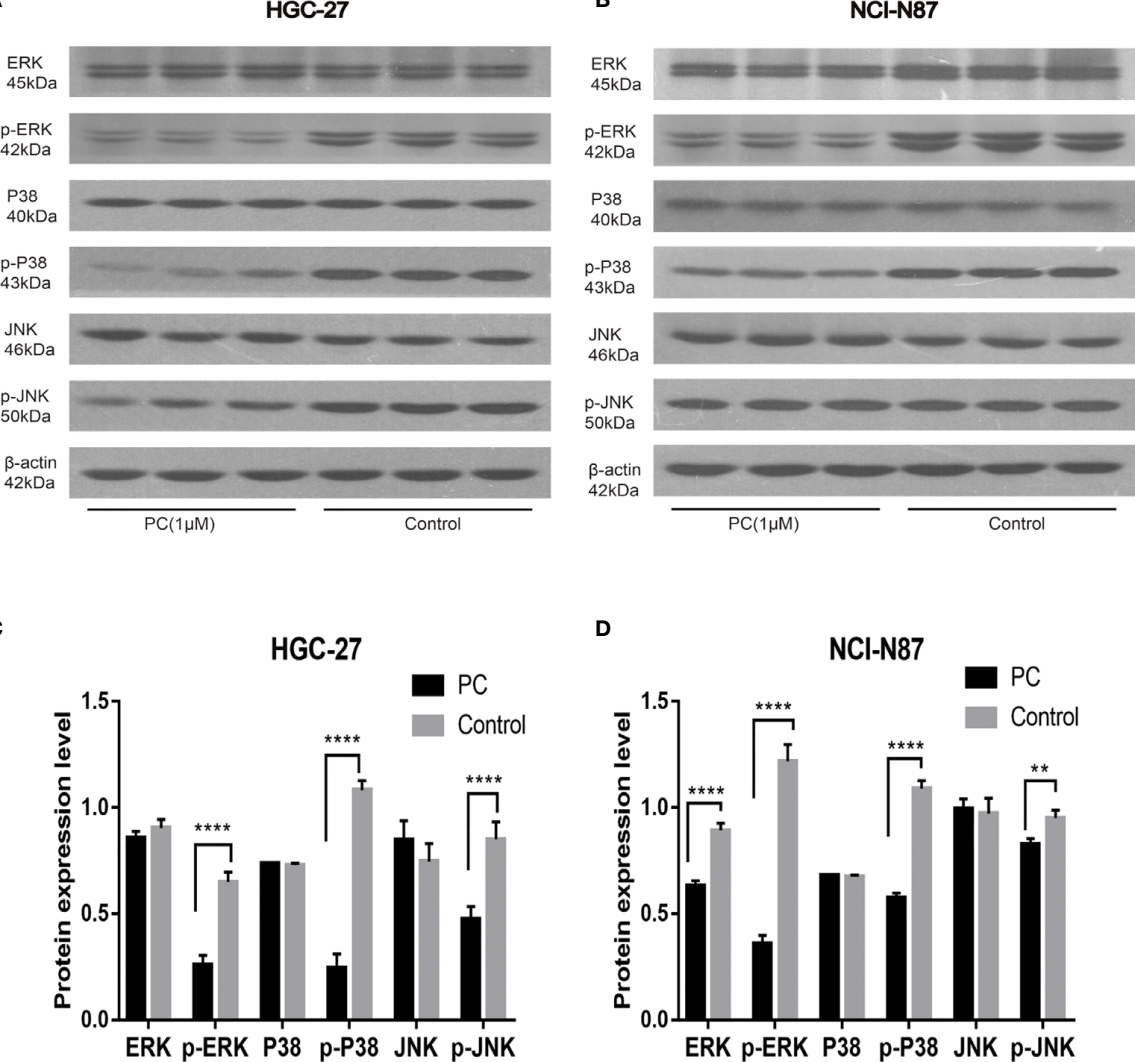
Effects of PA on the expression of proteins related to MAPK pathway in HGC-27 **(A, C)** and NCI-N87 **(B, D)** cells. (****P < 0.0001, **P < 0.01).

The network pharmacological findings indicate that the PI3K/AKT and MAPK signal pathways are likely to be linked to PA’s anticancer system, which regulates GC cell survival and proliferation. As a result, we used Western blot to detect the expression levels of PI3K, AKT, ERK, P38, JNK, and their phosphorylated products. PA significantly inhibited the phosphorylation levels of PI3K, AKT, ERK, P38, and JNK in NCI-N87 and HGC-27 cells as depicted in **Figures 10** and **11**. To summarize, PA’s effect on GC cells may be mediated by the PI3K/AKT and MAPK signaling pathways.

## 4 Discussion

The clinical application of TCM in China has a history of thousands of years. Most recently, TCM has attracted wide attention for possible use in the treatment of many diseases, including cancer. Studies have increasingly shown that some TCM remedies, including Chinese herbal medicine extracts, single TCM, and TCM formulations, may have anticancer effects (14–16). TCM has the features of multi-target, multi-pathway, and multi-action mechanisms, and its mechanism is difficult to clarify. However, in recent years, the network pharmacology produced by the combination of bioinformatics and pharmacology can clarify the mechanism of action of these herbs (17, 18). Therefore, we use the network pharmacology method for elucidating the PA mechanism in GC treatment. The *in vitro* experiments were used to further authenticate the potential mechanism of PA against GC.

In this study, PA was taken as the research object to study the mechanism of PA on GC from the point of view of network pharmacology. We sort out information from public databases for predicting the interaction between PA and its possible targets in GC. The network analysis through related software shows that PA could play a vital against GC *via* PI3K-AKT and MAPK signal pathways. In order to further verify this prediction, the therapeutic effects of PA on two kinds of GC cells were studied *in vitro*. The findings indicated that PA significantly inhibited the GC cell proliferation, and induced migration, apoptosis, and invasive activity in a dose-dependent manner.

Cell proliferation and cell cycle events are closely linked (19). Proliferating cell nuclear antigen (PCNA) has long been thought to be a strong indicator of cancer cell proliferation (20). The cyclin-dependent kinase activity is inhibited by p21, this, in turn, prevents cell cycle progression (21, 22). CyclinD1 has a positive regulatory effect on the cell cycle, and its overexpression can shorten the G1 phase of cells and lead to cell over-proliferation. Thus, it promotes the occurrence and development of tumors (23). Our study showed that the ratio of G0/G1 phase GC cells increased after PA treatment, while the ratio of G2/M phase and S phase cells decreased. Protein analysis indicated that the CyclinD1 and PCNA expression was down-regulated and the p21 expression was up-regulated. These results indicated that PA blocks the cell cycle of GC cells in the GO/G1 phase, thus inhibiting the proliferation of GC cells.

Apoptosis or programmed cell death is known to be related to various biological processes related to tumorigenesis (24). Apoptosis is triggered by different signal pathways, mainly the death receptor pathway and mitochondrial pathway (25). The apoptosis function of mitochondria is regulated by apoptosis regulatory proteins of the Bcl-2 family. Cell death and survival are determined by the levels of anti-apoptotic and pro-apoptotic proteins of the Bcl-2 family (26). As anti-apoptotic genes and pro-apoptotic genes, Bax and Bcl-2 are considered critical for apoptosis regulation (27). In addition, Bax forms apoptotic bodies, and when the expression level of caspase-3 is increased, caspase-3 is activated to induce apoptosis (28). After treatment with PA, apoptosis of HGC-27 and NCI-N87 cells was obviously induced. At the same time, the caspase-3 and Bax expression were significantly up-regulated, while the Bcl-2 expression was inhibited in the PA group. These results indicated that PA may play a tumor inhibitory role in the growth of GC cells.

Metastasis and invasion are the key steps in the occurrence and development of tumors. For patients with GC, cancer metastasis is often the principal cause of disease deterioration and death (29, 30). It has been confirmed that the expression levels of MMP-9 and MMP-2 in GC, especially in metastatic tumors, are noticeably higher than those in normal gastric mucosa (31, 32). In this study, the experiments of cell scratch and Transwell suggest that PA inhibited the migration and invasion of GC cells. More importantly, after PA treatment, the expression of MMP2 and MMP9 was down-regulated. We further proved that PA has the potential to inhibit the GC cell’s invasion and migration.

The proliferation and growth of tumors depend on the regulation and activation of many signal pathways. It is reported that the signaling pathway of PI3K/AKT is among the signal transduction pathways that frequently get activated in tumors, and participates in cell proliferation, cell cycle, migration, growth, apoptosis, and angiogenesis (33). In this study, the western blot indicated that the expression of p-Akt and p-PI3K decreased in a dose-dependent manner. The results mentioned above showed that the phosphorylation levels of PI3K and Akt proteins decreased in the cells treated with PA, which confirmed that PA inhibited the activity of the PI3K/Akt signal pathway *via* blocking the phosphorylation of Akt and PI3K proteins. Furthermore, the MAPK pathway plays a central role in tumorigenesis by promoting tumorigenesis and development (34). The MAPK signaling pathway, which is divided into JNK, p38, and ERKMAPKs, is also involved in the survival, proliferation, and apoptosis of cells (35). Therefore, we are interested in exploring whether PA has a protective effect on GC. The results indicated that the phosphorylation of JNK, p38, and ERK in the PA treatment group was considerably lower as compared to the control group. Our study shows that PA is an effective inhibitor of MAPK in GC cells.

This study explained the potential mechanism of PA on GC. However, the limitation of the current study was that we did not verify the mechanisms by which PA inhibited GC *in vivo*. Further studies using animal models are needed to investigate the effects of PA on GC progression. In future, more experiments are needed to provide support for our findings, and provide a theoretical basis for the development of new drugs.

## 5 Conclusions

In conclusion, our study investigated the possible mechanism of PA inhibiting GC through network pharmacology and experimental results. The findings suggest that can inhibit GC cell proliferation, induce apoptosis, and damage GC cell invasion and migration *via* regulations of the MAPK and PI3K-AKT signal pathways. The results of the combination of network pharmacology and experimental findings may provide an effective tool for exploring the action mechanism of traditional Chinese medicine.

## Data Availability Statement

The original contributions presented in the study are included in the article/supplementary material. Further inquiries can be directed to the corresponding author.

## Author Contributions

YS and LC designed the research, performed the experiments, analyzed data and wrote the paper; XW, JL, and JZ performed the experiments; BT, FZ, GL, and BH provided technical support and all of the reagent and chemical. BH, BT, and LZ reviewed the paper for intellectual content. YS and LC contributed equally to this work and should be considered as co-first authors. All authors contributed to the article and approved the submitted version.

## Funding

This project was funded by the National Natural Science Foundation of China (81202679) and the Scientific Research Program of Hebei Provincial Administration of traditional Chinese Medicine (2016053).

## Conflict of Interest

The authors declare that the research was conducted in the absence of any commercial or financial relationships that could be construed as a potential conflict of interest.

## Publisher’s Note

All claims expressed in this article are solely those of the authors and do not necessarily represent those of their affiliated organizations, or those of the publisher, the editors and the reviewers. Any product that may be evaluated in this article, or claim that may be made by its manufacturer, is not guaranteed or endorsed by the publisher.

## References

[1] Gullo I Grillo F Mastracci L Vanoli A Carneiro F Saragoni L. Precancerous Lesions of the Stomach, Gastric Cancer and Hereditary Gastric Cancer Syndromes. Pathologica (2020) 112(3):166–85. 10.32074/1591-951X-166 PMC7931572 33179620

[2] Chen W Li J Li C Fan HN Zhang J Zhu JS. Network Pharmacology-Based Identification of the Antitumor Effects of Taraxasterol in Gastric Cancer. Int J Immunopathol Pharmacol (2020) 34:2058738420933107. 10.1177/2058738420933107 32701378 PMC7378706

[3] Kurokawa Y Yang HK Cho H Ryu MH Masuzawa T Park SR. Phase II Study of Neoadjuvant Imatinib in Large Gastrointestinal Stromal Tumours of the Stomach. Br J Cancer (2017) 117(1):25–32. 10.1038/bjc.2017.144 28535156 PMC5520207

[4] Wang Z Qi F Cui Y Zhao L Sun X Tang W. An Update on Chinese Herbal Medicines as Adjuvant Treatment of Anticancer Therapeutics. Biosci Trends (2018) 12(3):220–39. 10.5582/bst.2018.01144 30012913

[5] Li YC Xian YF Ip SP Su ZR Su JY He JJ. Anti-Inflammatory Activity of Patchouli Alcohol Isolated From Pogostemonis Herba in Animal Models. Fitoterapia (2011) 82(8):1295–301. 10.1016/j.fitote.2011.09.003 21958968

[6] Zheng YF Xie JH Xu YF Liang YZ Mo ZZ Jiang WW. Gastroprotective Effect and Mechanism of Patchouli Alcohol Against Ethanol, Indomethacin and Stress-Induced Ulcer in Rats. Chem Biol Interact (2014) 222:27–36. 10.1016/j.cbi.2014.08.008 25168850

[7] Yu XD Xie JH Wang YH Li YC Mo ZZ Zheng YF. Selective Antibacterial Activity of Patchouli Alcohol Against Helicobacter Pylori Based on Inhibition of Urease. Phytother Res (2015) 29(1):67–72. 10.1002/ptr.5227 25243578

[8] Xie YC Tang F. Protective Effect of Pogostemon Cablin on Membrane Fluidity of Intestinal Epithelia Cell in Ischemia/ Reperfusion Rats After Ischemia/Reperfusion. Zhongguo Zhong Xi Yi Jie He Za Zhi (2009) 29(7):639–41. 19852300

[9] Jeong JB Choi J Lou Z Jiang X Lee SH. Patchouli Alcohol, an Essential Oil of Pogostemon Cablin, Exhibits Anti-Tumorigenic Activity in Human Colorectal Cancer Cells. Int Immunopharmacol (2013) 16(2):184–90. 10.1016/j.intimp.2013.04.006 23602914

[10] Yu J Qi Y Luo G Duan HQ Zhou J. Extraction and Analysis of the Essential Oil in Pogostemon Cablin by Enzymatic Hydrolysis and Inhibitory Activity Against Hela Cell Proliferation. Zhong Yao Cai (2012) 35(5):796–9. 23213744

[11] Lu X Yang L Lu C Xu Z Qiu H Wu J. Molecular Role of EGFR-MAPK Pathway in Patchouli Alcohol-Induced Apoptosis and Cell Cycle Arrest on A549 Cells *In Vitro* and *In Vivo*. BioMed Res Int (2016) 2016:4567580. 10.1155/2016/4567580 27830146 PMC5086517

[12] Zhao Y Fu G Wang J Guo M Yu G. Gene Function Prediction Based on Gene Ontology Hierarchy Preserving Hashing. Genomics (2019) 111:334–42. 10.1016/j.ygeno.2018.02.008 29477548

[13] Chen L Zhang YH Wang S Zhang Y Huang T Cai YD. Prediction and Analysis of Essential Genes Using the Enrichments of Gene Ontology and KEGG Pathways. PLoS One (2017) 12:e0184129. 10.1371/journal.pone.0184129 28873455 PMC5584762

[14] Li QY Hou CZ Yang LP Chu XL Wang Y Zhang P. Study on the Mechanism of Ginseng in the Treatment of Lung Adenocarcinoma Based on Network Pharmacology. Evid Based Complement Alternat Med (2020) 2020:2658795. 10.1155/2020/2658795 32802118 PMC7415121

[15] Wang Y Xu C Xu B Li L Li W Wang W. Xiaoai Jiedu Recipe Inhibits Proliferation and Metastasis of Non-Small Cell Lung Cancer Cells by Blocking the P38 Mitogen-Activated Protein Kinase (MAPK) Pathway. Med Sci Monit (2019) 25:7538–46. 10.12659/MSM.917115 PMC6792514 31590176

[16] Leigh AB Cheung HP Lin LZ Ng TB Lao L Zhang Y. Comprehensive and Holistic Analysis of HT-29 Colorectal Cancer Cells and Tumor-Bearing Nude Mouse Model: Interactions Among Fractions Derived From the Chinese Medicine Formula Tian Xian Liquid in Effects on Human Colorectal Carcinoma. Integr Cancer Ther (2017) 16(3):339–50. 10.1177/1534735416651969 PMC5759938 27261455

[17] Zhang Y Li X Xu X Yang N. Mechanisms of Paeonia Lactiflora in Treatment of Ulcerative Colitis: A Network Pharmacological Study. Med Sci Monit (2019) 25:7574–80. 10.12659/MSM.917695 PMC6798801 31594914

[18] Luo B Que ZJ Zhou ZY Wang Q Dong CS Jiang Y. Feiji Recipe Inhibits the Growth of Lung Cancer by Modulating T-Cell Immunity Through Indoleamine-2,3-Dioxygenase Pathway in an Orthotopic Implantation Model. J Integr Med (2018) 16(4):283–9. 10.1016/j.joim.2018.04.008 29752140

[19] Zhou RP Chen G Shen ZL Pan LQ. Cinobufacin Suppresses Cell Proliferation *via* miR-494 in BGC- 823 Gastric Cancer Cells. Asian Pac J Cancer Prev (2014) 15(3):1241–5. 10.7314/APJCP.2014.15.3.1241 24606447

[20] Czyzewska J Guzińska-Ustymowicz K Pryczynicz A Kemona A Bandurski R. Immunohistochemical Evaluation of Ki-67, PCNA and MCM2 Proteins Proliferation Index (PI) in Advanced Gastric Cancer. Folia Histochem Cytobiol (2009) 47(2):289–96. 10.2478/v10042-009-0042-y 19995716

[21] Di Cunto F Topley G Calautti E Hsiao J Ong L Seth PK. Inhibitory Function of p21Cip1/WAF1 in Differentiation of Primary Mouse Keratinocytes Independent of Cell Cycle Control. Science (1998) 280(5366):1069–72. 10.1126/science.280.5366.1069 9582119

[22] Dutto I Tillhon M Cazzalini O Stivala LA Prosperi E. Biology of the Cell Cycle Inhibitor P21(CDKN1A): Molecular Mechanisms and Relevance in Chemical Toxicology. Arch Toxicol (2015) 89(2):155–78. 10.1007/s00204-014-1430-4 25514883

[23] Hartwell LH Kastan MB. Cell Cycle Control and Cancer. Science (1994) 266(5192):1821–8. 10.1126/science.7997877 7997877

[24] Fesik SW. Promoting Apoptosis as a Strategy for Cancer Drug Discovery. Nat Rev Cancer (2005) 5(11):876–85. 10.1038/nrc1736 16239906

[25] Shang H Cao Z Zhao J Guan J Liu J Peng J. Babao Dan Induces Gastric Cancer Cell Apoptosis via Regulating MAPK and NF-κb Signaling Pathways. J Int Med Res (2019) 47(10):5106–19. 10.1177/0300060519867502 PMC6833375 31456462

[26] Lv L Liu B. Anti−Tumor Effects of Bakuchiol on Human Gastric Carcinoma Cell Lines Are Mediated Through PI3K/AKT and MAPK Signaling Pathways. Mol Med Rep (2017) 16(6):8977–82. 10.3892/mmr.2017.7696 28990045

[27] Ma L Zhu Y Fang S Long H Liu X Liu Z. Arenobufagin Induces Apoptotic Cell Death in Human Non-Small-Cell Lung Cancer Cells via the Noxa-Related Pathway. Molecules (2017) 22(9). 10.3390/molecules22091525 PMC6151516 28892004

[28] Kim SH Choo GS Yoo ES Woo JS Han SH Lee JH. Silymarin Induces Inhibition of Growth and Apoptosis Through Modulation of the MAPK Signaling Pathway in AGS Human Gastric Cancer Cells. Oncol Rep (2019) 42(5):1904–14. 10.3892/or.2019.7295 PMC6775811 31485597

[29] Hohenberger P Gretschel S. Gastric Cancer. Lancet (2003) 362(9380):305–15. 10.1016/S0140-6736(03)13975-X 12892963

[30] Krejs GJ. Gastric Cancer: Epidemiology and Risk Factors. Dig Dis (2010) 28(4-5):600–3. 10.1159/000320277 21088409

[31] Lim SC. Expression of Matrix Metalloproteinases and Its Inhibitor in Gastric Adenocarcinoma. Cancer Res Treat (2001) 33(3):199–206. 10.4143/crt.2001.33.3.199 26680786

[32] Lyu ZK Li CL Jin Y Liu YZ Zhang X Zhang F. Paeonol Exerts Potential Activities to Inhibit the Growth, Migration and Invasion of Human Gastric Cancer BGC823 Cells via Downregulating MMP−2 and MMP−9. Mol Med Rep (2017) 16(5):7513–9. 10.3892/mmr.2017.7576 PMC5865884 28944890

[33] Huang Y Lin J Yi W Liu Q Cao L Yan Y. Research on the Potential Mechanism of Gentiopicroside Against Gastric Cancer Based on Network Pharmacology. Drug Des Devel Ther (2020) 14:5109–18. 10.2147/DDDT.S270757 PMC7700081 33262572

[34] Liang Z Wu R Xie W Geng H Zhao L Xie C. Curcumin Suppresses MAPK Pathways to Reverse Tobacco Smoke-Induced Gastric Epithelial-Mesenchymal Transition in Mice. Phytother Res (2015) 29(10):1665–71. 10.1002/ptr.5398 26074474

[35] Gururajan M Dasu T Shahidain S Jennings CD Robertson DA Rangnekar VM. Spleen Tyrosine Kinase (Syk), A Novel Target of Curcumin, Is Required for B Lymphoma Growth. J Immunol (2007) 178(1):111–21. 10.4049/jimmunol.178.1.111 17182546

